# Preparation and Properties of Double Network Hydrogel with High Compressive Strength

**DOI:** 10.3390/polym14050966

**Published:** 2022-02-28

**Authors:** Bo Kang, Qingli Lang, Jian Tu, Jun Bu, Jingjing Ren, Bin Lyu, Dangge Gao

**Affiliations:** 1 National Engineering Laboratory for Exploration and Development of Low Permeability Oil and Gas Field, Xi’an 710018, China; kangbo1_cq@petrochina.com.cn (B.K.); langqingli_cq@petrochina.com.cn (Q.L.); tj4_cq@petrochina.com.cn (J.T.); bujun_cq@petrochina.com.cn (J.B.); 2 Oil &Gas Technology Research Institute of Changqing Oilfield Co, Xi’an 710018, China; 3 The 3rd Oil Production Plant of Changqing Oilfield Co., Yinchuan 750001, China; 4 The 10th Oil Production Plant of Changqing Oilfield Co., Qingcheng 745100, China; 5 College of Bioresources Chemical and Materials Engineering, Shaanxi University of Science and Technology, Xi’an 710021, China; 6 Xi’an Key Laboratory of Green Chemicals and Functional Materials, Shaanxi University of Science and Technology, Xi’an 710021, China

**Keywords:** p–DN hydrogel, compressive strength, viscosity, temperature and salt resistance, plugging

## Abstract

In this work, p–double network (p–DN) hydrogels were formed by the interpenetration of poly(2–acrylamide–2–methylpropanesulfonic acid–copolymer– acrylamide) microgel and polyacrylamide. The initial viscosity of prepolymer solution before hydrogel polymerization, mechanical properties, temperature and salt resistance of the hydrogels were studied. The results showed that the initial viscosity of the prepolymer was less than 30 mP·s, and the p–DN hydrogel not only exhibited high compressive stress (37.80 MPa), but the compressive strength of p–DN hydrogel could also reach 23.45 MPa after heating at 90 °C, and the compressive strength of p–DN hydrogel could reach 13.32 MPa after soaking for 24 h in the solution of 5W mineralization. In addition, the cyclic loading behavior of hydrogel was studied. The dissipation energy of p–DN hydrogel under 80% strain was 7.89 MJ/m^3^, which effectively dissipated energy. Meanwhile, p–DN hydrogel maintained its original form while breaking the pressure greater than 30 MPa, indicating excellent plugging performance.

## 1. Introduction

Most oil fields have entered the stage of medium and high water cut, and the water content of oil wells continues to rise, seriously affecting the production of crude oil [1,2,3]. Therefore, water plugging treatment is particularly important in technology and the economy [4,5,6,7]. Traditional water plugging agents, including resin, polymer cement and other materials, are not widely used due to the difficulty of filling micro cracks in medium and low permeability formations, difficult construction and high economic cost [8,9,10]. With the continuous development of oil fields and the complex geological conditions of the formation, existing plugging agents are insufficient.

Hydrogel is a hydrophilic, three–dimensional network material with chemical or physical crosslinking structure [11,12]. Due to the existence of its hydrophilic crosslinking network, the hydrogels can absorb a large amount of water and maintain a certain shape, and rapidly swell in water. Therefore, hydrogels can be applied to the field of water plugging in oil fields [13]. Although hydrogel has been used in the field of oil field water plugging [14,15], due to the uneven network structure of the traditional single network hydrogel plugging agent, it will cause permanent damage under external force, resulting in poor mechanical properties and the inability to meet the requirements of water–plugging strength [16,17]. In addition, the viscosity of the pre–polymerization solution before the formation of hydrogel is relatively high and cannot enter the underground smoothly to achieve the water shutoff effect during injection into the oil well. Therefore, in order to obtain a better plugging effect, it is imperative to develop hydrogel–plugging material with low viscosity and high strength.

Double–network (DN) hydrogel is formed by the mutual infiltration of two networks with opposite properties [18,19,20]. Compared with common interpenetrating network hydrogels, a synergistic effect exists between two network hydrogel polymer chains, so that the DN hydrogel has good mechanical properties, shear resistance, plugging performance, profile improvement and erosion resistance [21,22], which are widely used in many fields [23,24]. DN hydrogels have become one of the research hotspots of high–strength hydrogels because of their effective energy dissipation during fracture [25,26]. The two–step method is one of the most commonly used methods for preparing DN hydrogel [27]. However, the synthesis process involves swelling and diffusion, so it is difficult to control the monomer ratio of the first network and the second network, making it unable to form a specific shape and so on [28], which limits the application of DN hydrogel in oil field water plugging. Therefore, in order to decrease the initial viscosity of DN hydrogel, a microgel system consisting of first network microgel and second network monomer solution was proposed [29,30]. Its dispersed phase is a rigid microgel, and the continuous phase is soft matrix [31]. Microgel–enhanced DN hydrogel is also the hydrogel extension of DN hydrogel. It improves the two–step preparation of DN hydrogel due to the swelling caused by the inability to synthesize specific shape defects, and has mechanical properties comparable to the two–step preparation of DN hydrogel [32]. Therefore, we synthesized a hydrogel based on this microgel system, which can meet the requirements of water plugging.

**Scheme 1:**
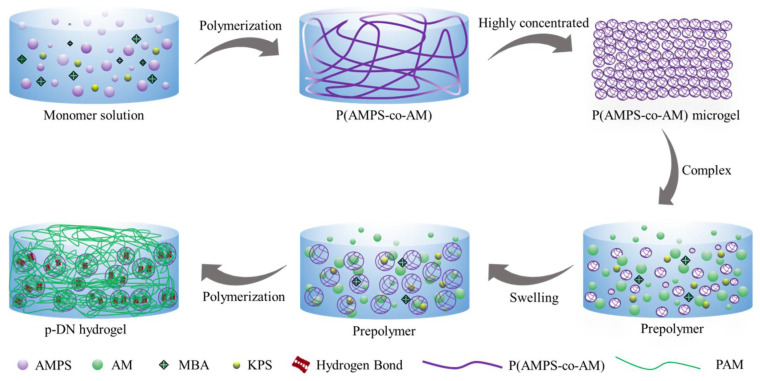
Flow chart of preparation of p–DN hydrogel.

Herein, p–DN hydrogel was synthesized using poly(2–acrylamide–2–methylpropanesulfonic acid–copolymer– acrylamide) (P(AMPS–co–AM)) microgel as the first network and polyacrylamide (PAM) as the second network (Scheme 1). The viscosity of the hydrogel prepolymer decreased with the addition of microgel system, and the introduction of AMPS enhanced the temperature and salt tolerance of hydrogels, which was beneficial to improving the mechanical properties of hydrogels. Meanwhile, the compressive strength, swelling capacity, dissipation energy, temperature and salt tolerance of p–DN hydrogel were also investigated. At the same time, a simulated plugging experiment of p–DN hydrogel was carried out. This kind of material is expected to be applied in the field of oil field water plugging.

## 2. Experimental

### 2.1. Materials

2–acrylamide–2–methylpropyl sulfonic acid (AMPS) was purchased in Xintai, N, N–methylenebis (acrylamide) (MBA) and acrylamide (AM) were purchased in Tianjin Komeo and potassium persulfate (KPS) was purchased in Tianjin Tianli.

### 2.2. Synthesis of P(AMPS–co–AM) Microgel

First, 17 wt% AMPS monomer, 1 wt% AM monomer, 0.6 wt% MBA crosslinking agent and 0.3 wt% KPS initiator were dissolved in water. After ultrasonication for 10 min in a supersonic wave, the solution was poured into the mold seal and then placed at 60 °C for 3 h to complete the polymerization to obtain the first network hydrogel. After the P(AMPS–co–AM) hydrogel was broken and dried, P(AMPS–co–AM) microgel was obtained.

### 2.3. Synthesis of p–DN Hydrogels

The AM monomer of 17.8 wt%, MBA crosslinking agent of 0.02 wt%, KPS initiator of 0.7 wt% and P(AMPS–co–AM) microgel of 0.5 wt% were dissolved in water, and the prepolymerization solution was obtained by stirring evenly. After the swelling equilibrium of P(AMPS–co–AM) microgel, the prepolymerization solution was sealed and placed in 60 °C for 4 h, and p–DN hydrogel was obtained. In addition, PAM hydrogel references were synthesized [33], and P(AMPS–co–AM)/PAM DN hydrogels were synthesized by the traditional two–step method.

### 2.4. Structural Characterization of Hydrogels

After freeze–drying the P(AMPS–co–AM) microgel and p–DN hydrogel, the structure was characterized by infrared spectroscopy and scanning.

### 2.5. Swelling Properties of Hydrogels

The hydrogel was freeze–dried and placed in water. Swelling hydrogel was removed regularly and surface water was removed. The quality of the hydrogel was measured to test the swelling ratio and the swelling ratio was calculated using the formula S = (m_t_ − m_o_)/m_o_×100% [33], where S (%) stands for swelling ratio, m_t_ (g) stands for mass of sample at time t and m_o_ (g) stands for dry weight of hydrogel after freeze–drying.

### 2.6. Performance Test of Hydrogel

(1) Prepolymerization liquid viscosity

The viscosity of prepolymer was measured by rotary viscometer (DV–2–PYO, Brookfield, Brookfield, WI, U.S.A).

(2) Mechanical property test

The compression strength of hydrogels was tested by high– and low–temperature servo tension machine (AI–7000–NGD, Gotwell, Dongguan, China).

(3) Temperature resistance

The DN hydrogels were immersed in water and placed in oven for 90 °C for a period of time. Then, the compression test was carried out.

(4) Thermal properties

The freeze–dried samples were tested by DSC at a heating rate of 10 °C/min under the protection of nitrogen at 40–120 °C(ci7800, X–Rite, Shanghai, China).

(5) Salt resistance

The hydrogels were placed in different salinity water, and their compressive strength was tested after soaking for a certain time.

(6) Simulation plugging experiment

Quartz sand with a density of 2.68 g/cm^3^ and a sand–filling tube with a size of 25.4 mm × 300 mm were selected. The displacement flow rate was set at a constant speed of 3 mL/min, and the experimental temperature was 25 °C. Under these conditions, the prepolymer was injected into the sand–filling tube. Finally, the sand–filling tube injected with prepolymer was placed in an oven at 60 °C for 6 h reaction to test its breakthrough pressure.

## 3. Results and Discussion

### 3.1. Prepolymerization Liquid Viscosity

**Figure 1:**
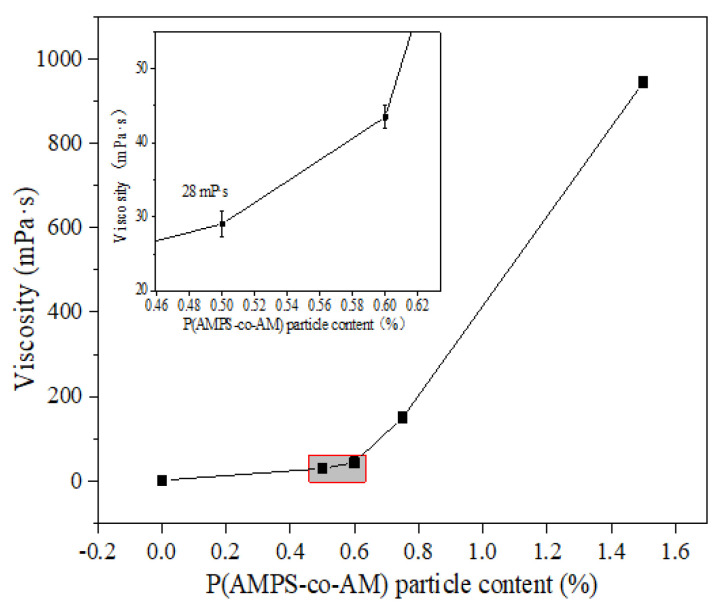
Effect of P(AMPS–co–AM) microgel content on the viscosity of prepolymerized liquid.

When using hydrogel as a plugging agent in oil wells, it was necessary to ensure that the hydrogel had good injectability, which meant that the hydrogel prepolymer had to have a low viscosity and could be successfully injected into the formation. Figure 1 shows the effect of P(AMPS–co–AM) microgel dosage on the viscosity of the prepolymer. As can be seen in Figure 1, the viscosity of the prepolymer increases sharply as the amount of P (AMPS–co–AM) microgel increases. When the dosage of P(AMPS–co–AM) microgel is 0.5%, the viscosity of the prepolymer is 28 mP·s, and when the dosage of microgel is 1.5%, the viscosity of mixed liquid reaches 980 mP·s. Therefore, in order to reduce the initial viscosity of the prepolymer solution, the P(AMPS–co–AM) microgel dosage is 0.5% at the later stage.

### 3.2. Structural Characterization

**Figure 2:**
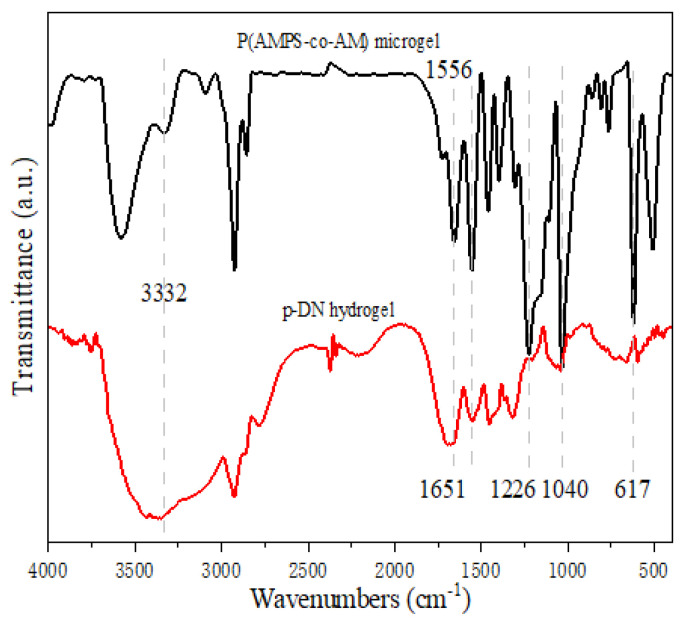
Infrared spectra of P(AMPS–co–AM) microgel and p–DN hydrogel.

Figure 2 shows the infrared spectra of P(AMPS–co–AM) microgel and p–DN hydrogel. As can be seen in Figure 2, in the curve of P(AMPS–co–AM), the strong absorption peak at 3332 cm^−1^ is attributed to the antisymmetric stretching vibration of N–H in the amide functional group. The peak at 1040 cm^−1^ and 617 cm^−1^ is the characteristic absorption peak of the sulfonic acid group (–SO_3_H), and the absorption peak at 1651 cm^−1^ is the carbonyl group (–C=O) in the amide functional group (amide I). At 1556 cm^−1^, there is an in–plane bending absorption peak of N–H in the amide functional group (amide Ⅱ), and at 1226 cm^−1^, there is a fitting peak of C–N stretching vibration and N–H bending vibration of the secondary amide, indicating that the polymer contained a sulfonic group, primary amide and secondary amide groups, which means that characteristic peaks of each monomer exist in the P(AMPS–co–AM) microgel. Compared with the infrared spectrum of P(AMPS–co–AM) microgel, the position of each characteristic peak of p–DN hydrogel is the same as those of P(AMPS–co–AM) microgel, which proved that the p–DN hydrogel was successfully synthesized.

**Figure 3:**
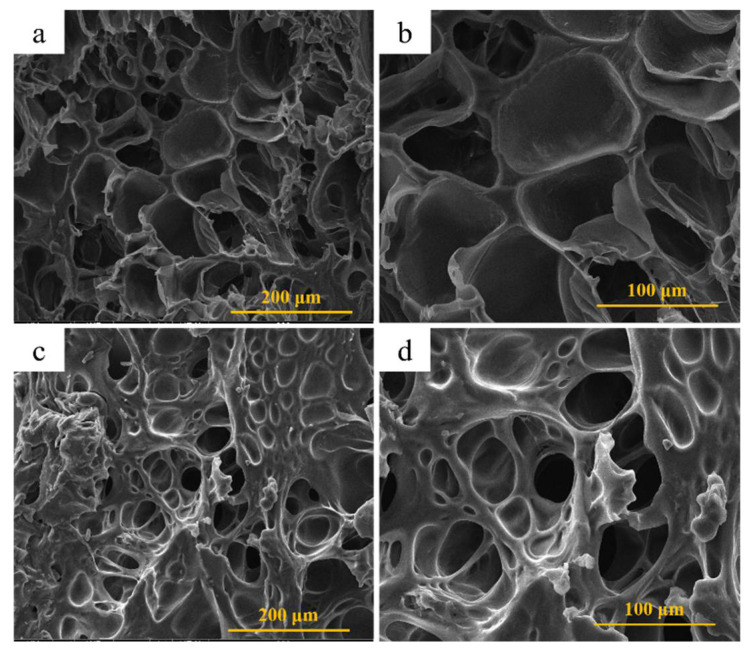
SEM photos of P(AMPS–co–AM) microgel (**a**,**b**) and p–DN hydrogel (**c**,**d**).

Figure 3 is a series of SEM photos of P(AMPS–co–AM) microgel and p–DN hydrogel. As shown in Figure 3a–b, P(AMPS–co–AM) microgel has larger surface holes and a random distribution of pore size. The main reason for the large pore size is that the AMPS side chain containing sulfonic groups, which have a large steric resistance and electrostatic repulsion between sulfonic acid groups. Figure 3c–d shows a SEM photo of the p–DN hydrogel. Compared with Figure 3a–b, the holes of the p–DN hydrogel are smaller and mostly closed. The main reason for the small and closed holes is that the AM monomer has no electrostatic repulsion, which could fill in the holes of P(AMPS–co–AM), reducing the electrostatic repulsion between sulfonic groups and making the holes of p–DN hydrogel smaller and denser.

### 3.3. Swelling Rate Test Results

**Figure 4:**
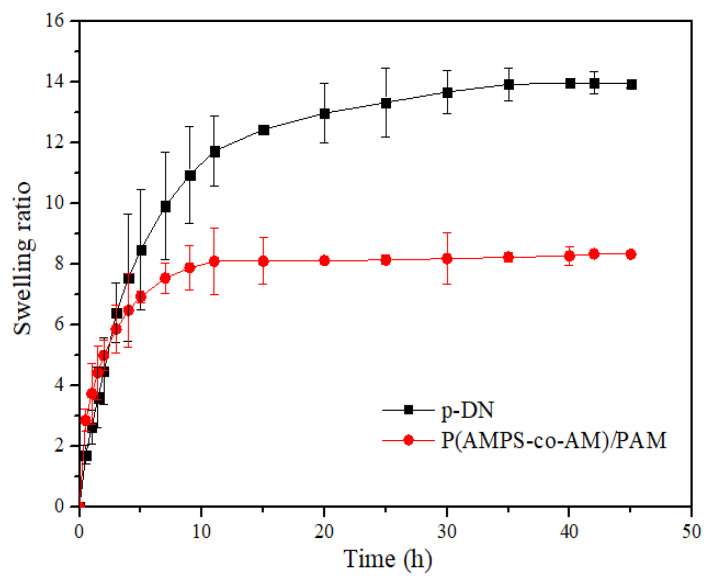
Swelling ratio of P(AMPS–co–AM)/PAM DN and p–DN hydrogels.

When hydrogels are used for water plugging in oil fields, on the one hand, they can be injected underground to plug the downhole cracks. On the other hand, the hydrophilic crosslinking network of the hydrogel will absorb the downhole water and enhance the oil recovery rate. Therefore, the amount of water absorbed by hydrogels (swelling rate) is one of the properties we need to investigate. The swelling properties of P(AMPS–co–AM)/PAM DN and p–DN hydrogels are shown in Figure 4. As can be seen in Figure 4, the swelling ratio of the p–DN hydrogel is slightly lower than that of the P(AMPS–co–AM)/PAM DN hydrogel in the first 2 h, and the swelling ratio of p–DN hydrogel is much higher than that ofthe P(AMPS–co–AM)/PAM DN hydrogel after 2 h. Meanwhile, the swelling of the p–DN hydrogel and P(AMPS–co–AM)/PAM DN hydrogel arrive at equilibrium after 40 h, at which point the equilibrium swelling ratio of p–DN hydrogel reaches 13.93, and that of the P(AMPS–co–AM)/PAM double–network hydrogel is 8.35. This phenomenon could be interpreted as follows: that in P(AMPS–co–AM)/PAM DN hydrogel, the complete P(AMPS–co–AM) rigid network acted in support of the DN hydrogel. When the swelling ratio reached 8.35, the P(AMPS–co–AM) network reached the fully extended state, which prevented the further swelling of the DN hydrogel. The p–DN hydrogel was equivalent to artificially breaking P(AMPS–co–AM)’s rigid network, so that P(AMPS–co–AM) exists in the dispersed phase in the p–DN hydrogel, and there was no complete P(AMPS–co–AM) rigid network to limit the swelling of the hydrogel. Therefore, the equilibrium swelling ratio of p–DN hydrogel is larger than that of the P(AMPS–co–AM)/PAM DN hydrogel.

### 3.4. Compression Strength of the Hydrogels

**Figure 5:**
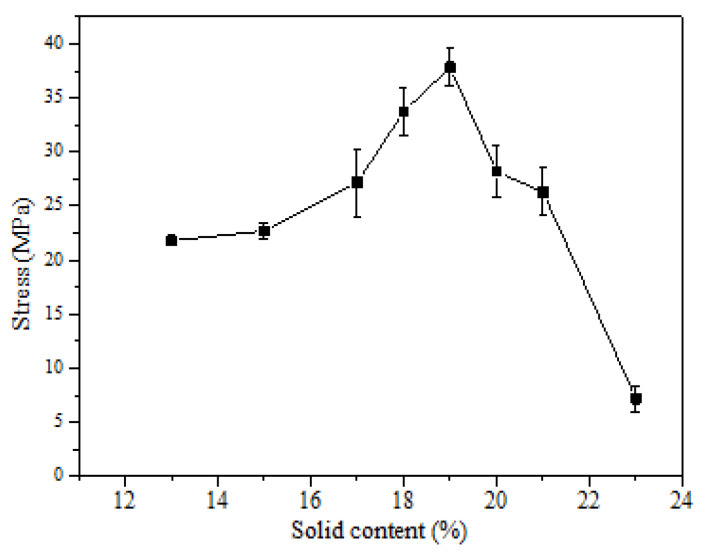
Effect of solid content on compression properties of p–DN hydrogel.

Figure 5 represents the effect of solid content on the compressive strength of p–DN hydrogels. It can be seen from the diagram that the compressive strength of the p–DN hydrogel first increases and then decreases with the increase in solid content. When the solid content is 19%, it reaches a maximum of 37.80 MPa, which meets the requirements of the mechanical properties of a water–plugging agent for medium– and high–water–cut oil wells. When the solid content reaches 23%, the compressive strength of the p–DN hydrogel is 7.18 MPa, which is nearly 30 MPa lower than that of a hydrogel with a solid content of 19%. The reduction in compressive strength might be due to the amount of P(AMPS–co–AM) microgel. The higher solid content means a larger proportion of PAM. The excessive PAM molecular chain hinders the stress transfer efficiency between the second network and the first network, thus affecting the compression strength of the p–DN hydrogel.

### 3.5. Dissipation Energy of Hydrogels

**Figure 6:**
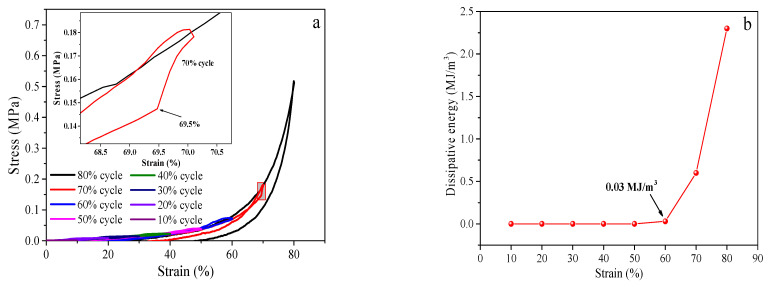

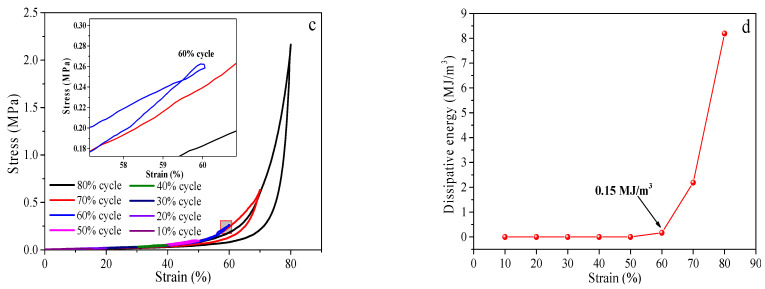
Cyclic compression and dissipation energy of hydrogel under different strains (cyclic compression (**a**) and dissipative energy (**b**) of PAM hydrogel; cyclic compression (**c**) and dissipative energy (**d**) of p–DN hydrogel).

Figure 6a and b show the cyclic compression and dissipation energy of PAM hydrogel under 10–80% strain. It can be seen in Figure 6a that when the cyclic compressive strain is 60%, a hysteresis loop begins to appear, indicating that dissipation energy begins to occur when the strain reaches 60%, and the dissipation energy of PAM hydrogel at 60% strain is 0.03 MJ/m^3^ in Figure 6b. Figure 6c,d, show the cyclic compression and dissipation energy of the p–DN under 10–80% strain. In Figure 6c, the p–DN hydrogel begins to produce a hysteresis loop when the cyclic compressive strain is 60%, and the dissipation energy at 60% strain is 0.15 MJ/m^3^, which is larger than that of the PAM hydrogel under 60% strain. This is mainly because the PAM network and the P(AMPS–co–AM) network form an interpenetrating network structure. The PAM chain transfered stress to the P(AMPS–co–AM) network during compression, which resulted in a partial fracture of the P(AMPS–co–AM) chain. Therefore, the p–DN hydrogel has a higher dissipation energy under the same strain.

**Figure 7:**
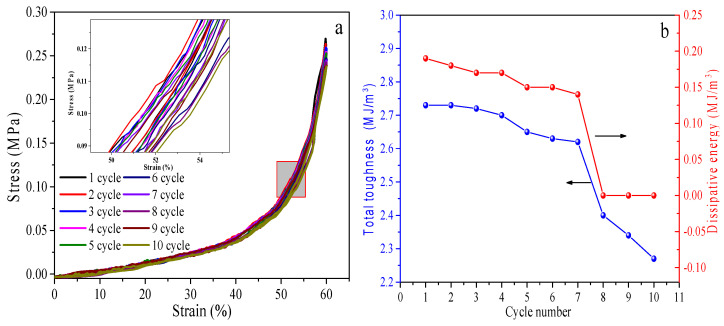

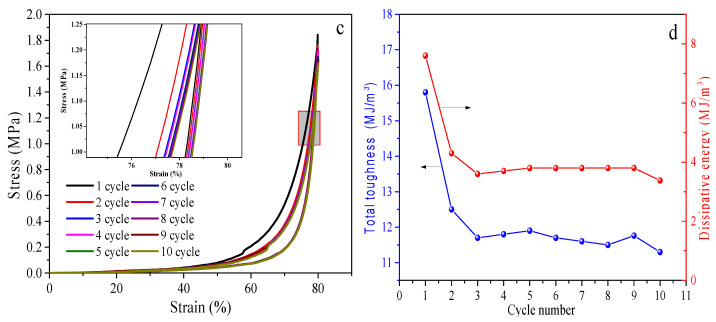
Ten cycles compression stress–strain curve and dissipated energy of p–DN hydrogel ((**a**): 60% strain; (**b**): dissipated energy at 60% strain; (**c**): 80% strain; (**d**): dissipated energy at 80% strain).

The cyclic compression curves and dissipation energy of the p–DN hydrogels at 60% and 80% strain are shown in Figure 7. It is observed in Figure 7a that there is no obvious hysteresis loop in ten cycles, which indicates that the PAM network is less broken when the strain is 60%. It can be seen in Figure 7b that as the number of cycles increases, the dissipation energy of the p–DN hydrogel decreases from 0.2 MJ/m^3^ to 0 MJ/m^3^, and the toughness continues to decrease, indicating that when the strain is 60%, there is no more PAM molecular chain fracture after seven cycles, and the toughness keeps decreasing. This is mainly due to the decrease in physical entanglement between PAM and P(AMPS–co–AM) during cyclic compression.

As seen in Figure 7c, the p–DN hydrogel generates a large hysteresis loop in the first cycle compression, and the dissipation energy of the first cycle compression reaches 7.89 MJ/m^3^, whereas the hysteresis loop size was basically the same in the second to tenth cycles. This is mainly because the P(AMPS–co–AM) network was broken under 80% strain and absorbed most of the energy as the “sacrifice bond”, whereas the dissipation energy in the second to tenth cycles mainly came from the broken P(AMPS–co–AM) network and the dissociation of physical entanglement between the PAM and P(AMPS–co–AM) network.

### 3.6. Temperature and Salt Resistance of p–DN Hydrogel

**Figure 8:**
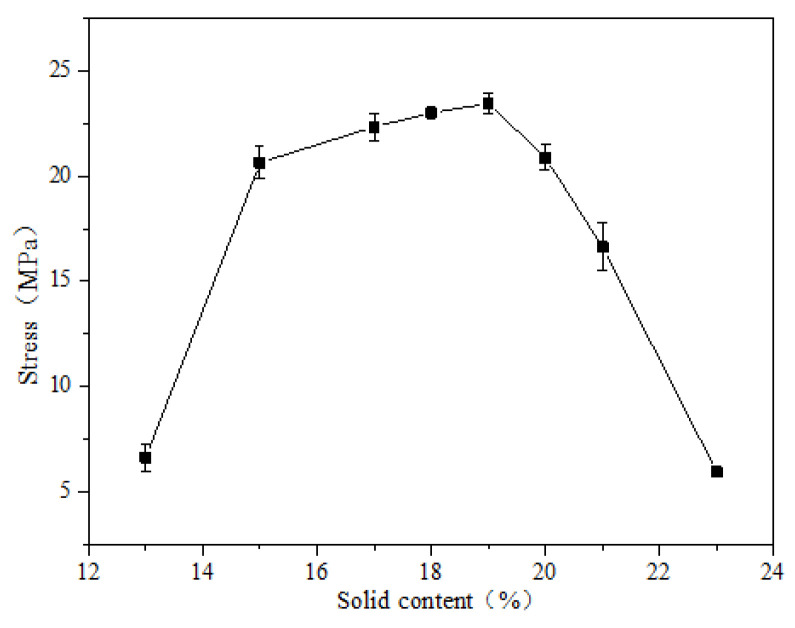
Temperature resistance of p–DN hydrogel.

To determine the applicable scope of p–DN hydrogels, hydrogels with different solid content were placed in an aqueous solution at 90 °C and then tested for compressive strength. Figure 8 shows the temperature resistance of p–DN hydrogels with different solid content. Combined with Table 1, the compressive strength of the p–double network hydrogel after high temperature treatment first increases and then decreases with the increase in solid content. When the solid content is between 15% and 21%, the compression strength of the p–DN hydrogels can reach more than 15 MPa after heating, and when the solid content is 19%, the compression modulus and toughness of the p–DN hydrogels reaches maximum after 10 h in 90 °C water, and the compression strength can still reach 23.45 MPa. Compared with that before heating, it only decreases by 37.9%, indicating that the p–DN hydrogel has the best temperature resistance when solid content is 19%.

**Figure 9:**
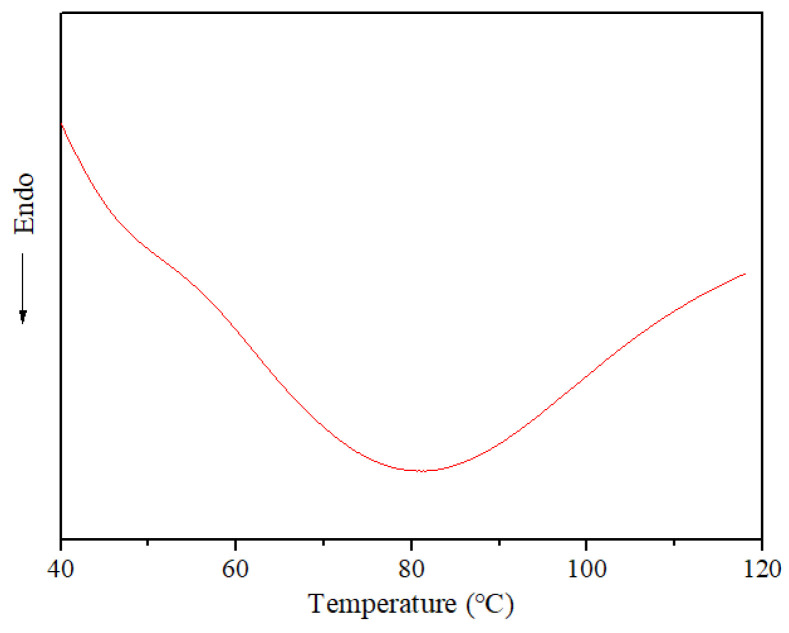
DSC characterization of p–DN hydrogel.

To further investigate the thermal properties of p–DN hydrogels, DSC characterization was performed. Figure 9 shows the DSC curve of the p–DN hydrogel. In the range of 40–120 °C, there is an endothermic peak around 80 °C, which can be attributed to the evaporation of water in the gel system. The main reason is that the freeze–dried samples used in the testing process had a porous structure and hydrophilic properties. Therefore, the freeze–dried samples absorb more moisture in the air, making the gel retain a small amount of water.

Groundwater has a certain salt concentration. Therefore, when the prepared prepolymerization solution enters into the underground, the groundwater containing salt could affect polymerization. So, the effect of groundwater with different salt concentration on the mechanical properties of the hydrogel was investigated.

**Figure 10:**
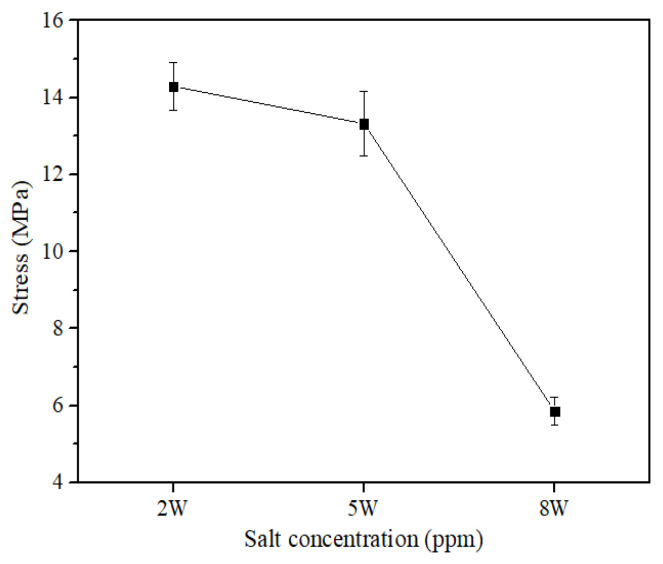
Effect of salt concentration on compressive stress of p–DN hydrogel.

The compressive strength of the p–DN hydrogel under different salt concentrations is shown in Figure 10. In combination with Table 2, it can be seen that as the salt concentration increases, the compressive strength gradually decreases, and the compressive strength decreases to 5.86 MPa at a salt concentration of 8 W. The main reason for the decrease in compressive strength is that the hydrogel in the water system soaked for a long time and then swelled to produce micro cracks. Meanwhile, the salt solution could penetrate into the micro cracks and aggravate the growth of micro cracks under the condition of high salt content.

### 3.7. Sealing Experiment Evaluation of p–DN Hydrogel

In order to simulate the practical application environment of the p–DN hydrogel, a sand tube test was carried out. Firstly, after the quartz sand was loaded into the sand– filling pipe, it was treated with saturated water, saturated oil and water flooding in order to simulate the formation environment. Subsequently, the hydrogel prepolymer was injected into the sand–filling pipe, and the sand filling pipe injected with the prepolymer was put into the oven at 60 °C to polymerize and test the pressure of breaking the hydrogel.

**Figure 11:**
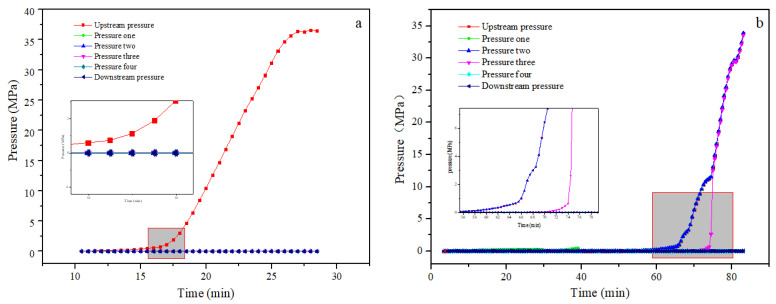
Experimental curves of forward breakthrough (**a**) and reverse breakthrough pressure (**b**).

We used two directions of pressure to test the hydrogel (the breakthrough pressure was defined from left to right as positive breakthrough), as shown in Figure 11. Figure 11a shows the pressure versus time curve in the forward breakthrough pressure experiment. It can be seen in Figure 11 that the upstream pressure of the sand–filling pipe increases with the increase in time, and when the time increases to 27 min, the upstream pressure increases to 36 MPa. In addition, the pressure at several pressure measurement points and the downstream pressure of the sand–filled pipe almost did not rise, and the curves overlap, indicating that the breakthrough pressure did not affect any pressure points. There was no water outflow from the end of the sand–filled pipe, indicating that the p–DN hydrogel had not yet been broken. Figure 11b shows the pressure change curve with time in the reverse breakthrough pressure experiment. With the increase in time, the pressure of the upstream of the sand–filling pipe and the pressure measuring point rise gradually. Moreover, the pressure three curve rises, but the pressure four curve does not change, indicating that the breakthrough pressure has reached pressure three, and there was still no water drop at the end of the sand–filled pipe, which further indicates that the p–DN had not broken through. When the pressure increased to 33 MPa, there was still no water dropout from the end of the sand–packed pipe, indicating that the p–DN had not broken through. The simulated plugging test showed that the hydrogel maintained its original gel morphology even at a breakthrough pressure of more than 30 MPa. Compared with the traditional hydrogel water plugging material, its strength increased greatly, proving that p–DN hydrogel has excellent plugging performance.

## 4. Conclusions

p–DN hydrogels were synthesized using a P(AMPS–co–AM) microgel as the first network and a PAM as the second network, which had low initial viscosity, high compression strength and temperature and salt resistance when entered into the ground. The compressive strength of the p–DN hydrogel was 37.80 MPa, the fracture strain was greater than 90%, the dissipation energy was 7.89 MJ/m^3^ and the compressive strength remained at 23.45 MPa under a high–temperature (90 °C) treatment and 13.32 MPa under a high–salinity treatment. When the pressure exceeded 30 MPa, the hydrogel was not destroyed and had an excellent plugging effect. This work provides a kind of hydrogel with high strength and certain temperature and salt resistance. This kind of material is expected to be applied to the field of water plugging in oil fields, and the application of hydrogel in water plugging could be realized.

## Figures and Tables

**Table 1:** Temperature resistance of p–DN hydrogels.

Solid Content	Compressive Strength (MPa)	Strain (%)
13%	6.21 ± 0.64	>90
15%	21.23 ± 0.78	>90
17%	22.32 ± 0.64	>90
18%	23.07 ± 0.14	>90
19%	23.45 ± 0.49	>90
20%	21.51 ± 0.58	>90
21%	16.84 ± 1.12	>90
23%	5.42 ± 0.23	>90

**Table 2:** Salt tolerance of p–DN hydrogels.

Salt Concentration (ppm)	Compressive Strength (MPa)	Strain (%)
2 W	14.29 ± 0.62	>90
5 W	13.32 ± 0.83	>90
8 W	5.86 ± 0.35	>90
